# Resveratrol ameliorates polycystic ovary syndrome via transzonal projections within oocyte-granulosa cell communication

**DOI:** 10.7150/thno.67167

**Published:** 2022-01-01

**Authors:** Mingyue Chen, Chengyong He, Kongyang Zhu, Zihan Chen, Zixiao Meng, Xiaoming Jiang, Jiali Cai, Chunyan Yang, Zhenghong Zuo

**Affiliations:** 1 State Key Laboratory of Cellular Stress Biology, United Diagnostic and Research Center for Clinical Genetics, Women and Children's Hospital, School of Life Sciences, Xiamen University, Xiamen, People's Republic of China.; 2 Reproductive Medicine Hospital, Xiamen University Affiliated Chenggong Hospital.

**Keywords:** PCOS, oocyte-GC communication, TZP assembly, resveratrol, CaMKIIβ

## Abstract

**Rationale:** Polycystic ovary syndrome (PCOS) is closely linked to follicular dysplasia and impaired bidirectional oocyte-granulosa cell (GC) communication. Given that PCOS is a heterogeneous, multifactorial endocrine disorder, it is important to clarify the pathophysiology of this ovarian disease and identify a specific treatment.

**Methods:** We generated PCOS rat models based on neonatal tributyltin (TBT) exposure and studied the therapeutic effect and mechanism of resveratrol (RSV), a natural plant polyphenol. Transcriptome analysis was conducted to screen the significantly changed pathways, and a series of experiments, such as quantitative real-time polymerase chain reaction (PCR), Western blot and phalloidin staining, were performed in rat ovaries. We also observed similar changes in human PCOS samples using Gene Expression Omnibus (GEO) database analysis and quantitative real-time PCR.

**Results:** We first found that injury to transzonal projections (TZPs), which are specialized filopodia that mediate oocyte-GC communication in follicles, may play an important role in the etiology of PCOS. We successfully established PCOS rat models using TBT and found that overexpressed calcium-/calmodulin-dependent protein kinase II beta (CaMKIIβ) inhibited TZP assembly. In addition, TZP disruption and *CAMK2B* upregulation were also observed in samples from PCOS patients. Moreover, we demonstrated that RSV potently ameliorated ovarian failure and estrus cycle disorder through TZP recovery via increased cytoplasmic calcium levels and excessive phosphorylation of CaMKIIβ.

**Conclusions:** Our data indicated that upregulation of CaMKIIβ may play a critical role in regulating TZP assembly and may be involved in the pathogenesis of PCOS associated with ovarian dysfunction. Investigation of TZPs and RSV as potent CaMKIIβ activators provides new insight and a therapeutic target for PCOS, which is helpful for improving female reproduction.

## Introduction

Polycystic ovary syndrome (PCOS) is one of the major causes of ovulatory dysfunction, affecting 6%-20% of women of reproductive age worldwide 1, 2. Women with PCOS exhibit a wide range of clinical symptoms, including clinical or biochemical hyperandrogenism, oligo- or anovulation caused by cessation of follicular growth and polycystic ovarian changes 3, 4. Despite the high incidence of PCOS and its adverse effects on women's health, the exact etiology and pathogenesis of PCOS are poorly understood. Although PCOS is familial in most cases, environmental exposure is also considered to play an essential role in PCOS pathogenesis 5, 6. Endocrine-disrupting chemicals (EDCs), which comprise a group of chemical compounds, interfere with endocrine system hormone regulation and thus influence human reproduction and development 7. Thus, clarifying how EDCs induce PCOS is crucial.

Tributyltin (TBT), a typical EDC, is a persistent organotin compound that is widely applied as a biocide in antifouling paints 8. Due to its long half-life and enrichment in the food chain, humans are at a high risk of TBT exposure 9. Even in recent years, high TBT levels have been reported in several studies 10-14. The maximum concentration of butyltin compounds in fish livers from the Polish coast of the Baltic Sea was 1131 ng Sn g^(-1)^ dry weight in 2016 15. A survey reported that 50-400 nM TBT was detected in human blood in 2018 16. Our previous study found that exposure to environmentally relevant levels of TBT leads to PCOS in rats 17, but the pathogenic mechanism and effective solutions need to be investigated.

PCOS is associated with a high prevalence of type 2 diabetes mellitus, metabolic syndrome, hyperandrogenism and cardiovascular disease 18. Anti-androgen therapy or suppression of ovarian function is often associated with adverse effects and is considered clinically unacceptable by patients 19. Therefore, the development of other therapies, especially natural herbal remedies, is desperately needed for the clinical treatment of PCOS. Resveratrol (3,5,4-trihydroxystilbene, RSV), a natural polyphenolic phytoalexin, is a polyphonic compound found in grapes, red wine, and peanuts 20. Accumulating evidence indicates that RSV possesses various biological effects, including antioxidant, anti-inflammatory and antithrombotic effects 21, 22. Treatment with RSV ameliorates the elevated number of small antral and atretic follicles and the decreased number of mature follicles in PCOS rats, revealing the effect of RSV on the maintenance of folliculogenesis 23. Although RSV has shown potential benefits in the treatment of PCOS 24, the potential mechanisms need to be completely elucidated.

In the present study, we found that neonatal exposure to environmentally relevant levels of TBT leads to PCOS-like syndrome in adult rats. Through transcriptome and Gene Expression Omnibus (GEO) database analyses, we demonstrated that transzonal projection (TZP) damage in oocyte-granulosa cell (GC) bidirectional communication plays an essential role in the pathogenetic process of PCOS. We further showed that RSV recovered the inward flow of calcium ions and activated excess CaMKIIβ to release more free monomeric actin, thus enabling TZP synthesis. Our work provides novel insight into the pathogenetic mechanism of PCOS and demonstrates that RSV represents a potential natural remedy for the treatment of PCOS, which is meaningful for improving female reproduction.

## Results

### RSV ameliorated TBT-induced impaired estrus cycles and sexual hormone disturbances in rats

**Figure 1:**
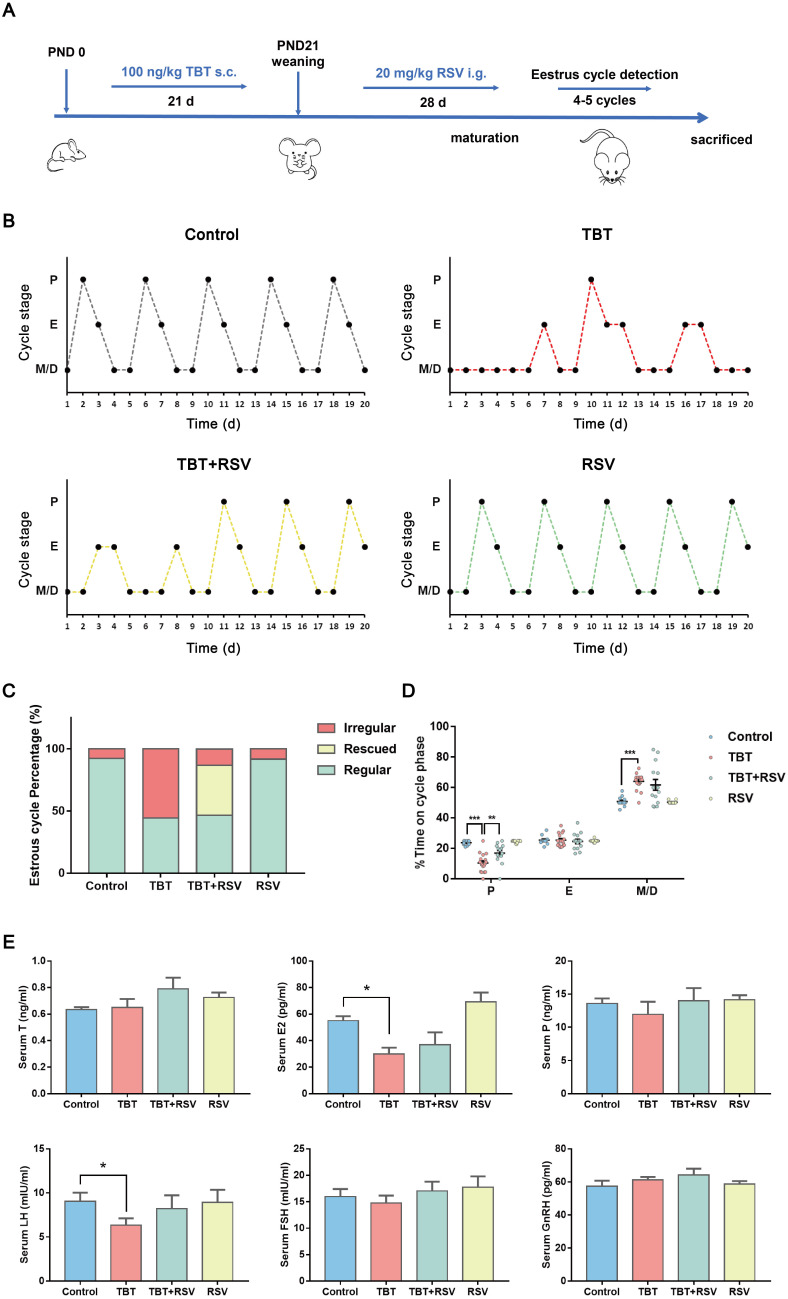
** Protective effects of RSV on impaired estrus cycles and sexual hormone disturbance induced by TBT. A,** Schematic illustration of the experimental design. s.c.: subcutaneous injection, i.g.: intragastric administration, PND: postnatal day. **B,** Representative estrus cyclicity of adult (2-3 months) rats over 16 consecutive days. M/D: metestrus/diestrus phase, P: proestrus, E: estrus. **C,** The proportions of regular, irregular and rescued estrus cycles; rescued cycles were defined as regular estrus cycles observed at the later part of the study in rats in the TBT + RSV group. **D,** Quantitative analysis of estrus cyclicity in adult rats (Control, n = 12; TBT, n = 18; TBT + RSV, n = 14; RSV, n = 11). **E,** The hormone levels of adult rats (3 months) in estrus (control, n = 8; TBT, n = 9; TBT + RSV, n = 9; RSV, n = 9). Data are presented as the mean ± SEM. Comparisons between two groups were analyzed using unpaired Student's t-test and IBM SPSS Statistics 19 (IBM, USA). Statistical analyses were performed using the GraphPad Prism 7 program, and *p* ≤ 0.05 was considered statistically significant, *p ≤ 0.05, **p ≤ 0.01, ***p ≤ 0.001.

In a previous study, we revealed that neonatal exposure to environmentally relevant levels of TBT leads to PCOS-like symptoms in adult rats 17. To determine whether the natural product RSV provides a protective effect against TBT-induced PCOS, we treated newborn female SD rats with 100 ng/kg TBT or vehicle by subcutaneous injection for 21 days until weaned followed by treatment with or without 20 mg/kg RSV by gavage for 28 days. We assessed the estrus cycle for 20 consecutive days in 2- to 3-month-old rats (Fig. 1A) and found that approximately half of TBT-treated rats displayed irregular estrus cycles compared to control group rats, but rats treated with RSV after TBT exposure tended to have improved estrus cycles (Fig. 1B, C). As shown in Figure 1D, the proportion of time spent in estrus minimally differed among all the groups, and the proportions of time spent in metestrus and diestrus showed large individual variations between the TBT-treated group and the combined treatment group. The proportion of time spent in proestrus, the time when follicle growth accelerates, was significantly decreased in the TBT-treated group, whereas RSV treatment significantly extended the time in proestrus.

To determine the internal exposure dose, total tin concentrations in liver samples were detected, revealing increases in the TBT and combined groups (Fig. S1). To investigate the effects on sexual hormones, we collected rat sera from the submandibular vein after 21 days of TBT exposure and from rats under deep terminal anesthesia by cardiac puncture before sacrifice. TBT-exposed infant rats showed higher levels of progesterone (P), luteinizing hormone (LH) and follicle-stimulating hormone (FSH) than control rats (Fig. S2A). Sex steroid action that occurs too early, as observed in precocious puberty, has been identified as a prepubertal risk factor for the development of PCOS 25, 26. We identified the day of vaginal patency acquisition (sexual maturation) and found that the TBT-exposed rats were more likely to be premature (Fig. S2B). However, LH and estradiol (E2) levels were significantly decreased in adult rats treated with TBT and were attenuated by treatment with RSV after maturation. The levels of testosterone (T), P, FSH and gonadotropin-releasing hormone (GnRH) showed no significant differences (Fig. 1E), indicating that TBT-treated rats showed no hyperandrogenism or obvious disruption of the hypothalamic-pituitary-gonadal axis. Above all, TBT-exposed rats displayed disordered estrus cycles and sexual hormone disturbances, both of which were remedied by RSV treatment.

### RSV ameliorated TBT-induced ovarian failure

**Figure 2:**
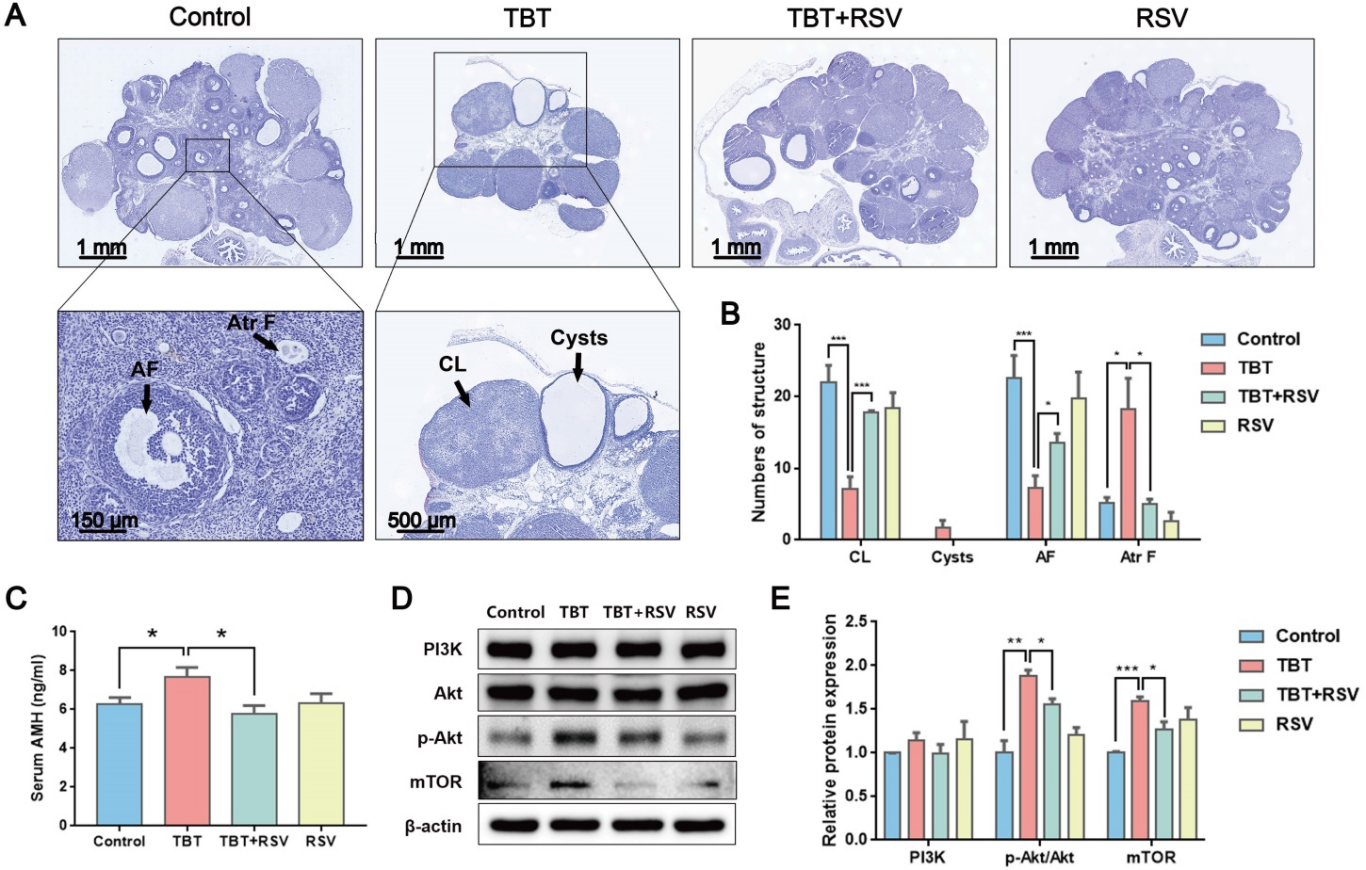
** RSV ameliorated TBT-induced ovarian failure. A,** Representative H&E staining images of rat ovaries. CL: corpus luteum, AF: antral follicles, Atr F: atretic follicles. **B,** Follicle counting results for ovaries from all four groups (Control, n = 5; TBT, n = 6; TBT + RSV, n = 4; RSV, n = 3). **C,** Plasma AMH levels in adult (3 months old) diestrus females in estrus (Control, n = 8; TBT, n = 9; TBT + RSV, n = 9; RSV, n = 9). **D,** Expression of PI3K, Akt, p-Akt, and mTOR protein in ovaries. **E,** The results are expressed as the fold change in the optical density of a target protein, and β-actin expression served as the control. The mean protein expression of the control is designated as 1 in the graph. Data are presented as the mean ± SEM (n = 3-6). Data were compared between two groups with Student's t-test, *p ≤ 0.05, **p ≤ 0.01, ***p ≤ 0.001.

As the basic functional unit of female reproduction, the ovarian follicle is composed of germline oocytes and follicular somatic cells. Folliculogenesis is an accurate and orderly physiological process involving many endocrine, paracrine and autocrine factors in mammals 27. Histological analysis of the ovarian follicles revealed that the TBT group had fewer corpus luteum and antral follicles, ovarian cysts, and more atretic follicles than the controls. These PCOS-like systems were markedly attenuated by RSV (Fig. 2A, B). Anti-Müllerian hormone (AMH), a polypeptide of the transforming growth factor beta (TGF-β) family, is exclusively secreted by GCs of preantral and small antral follicles and plays an important role in folliculogenesis. Consistent with excessive ovarian follicles, AMH is elevated in women with PCOS compared with healthy women 28. In this study, TBT-exposed rats had a significantly increased AMH level. However, after RSV treatment, AMH decreased to normal levels (Fig. 2C). The phosphatidylinositol 3-kinase (PI3K) pathway in oocytes regulates the activation of primordial follicles. Under the control of protein kinase B (Akt) and mammalian target of rapamycin (mTOR) signaling, selective primordial follicles develop into the primary stage, and activated follicles not selected for further development undergo atresia 29. The expression of proteins involved in the PI3K signaling pathway was detected to analyze the activation of primordial follicles in ovaries. We found that the relative PI3K level was not significantly different among the control and treatment groups. However, Akt phosphorylation levels were significantly increased in TBT-exposed rats, and a significant increase in mTOR was subsequently noted. RSV attenuated TBT-induced Akt phosphorylation and mTOR expression levels (Fig. 2D, E), suggesting that RSV has a protective effect on TBT-induced primordial follicle pool exhaustion by blocking the phosphorylation of members of the PI3K/Akt/mTOR pathway. Accordingly, we deduced that TBT could lead to follicular dysplasia and result in ovulation disorders and polycystic ovaries, which were ameliorated by RSV treatment.

### RSV recovered the proliferative arrest and apoptosis of granulosa cells caused by TBT

**Figure 3:**
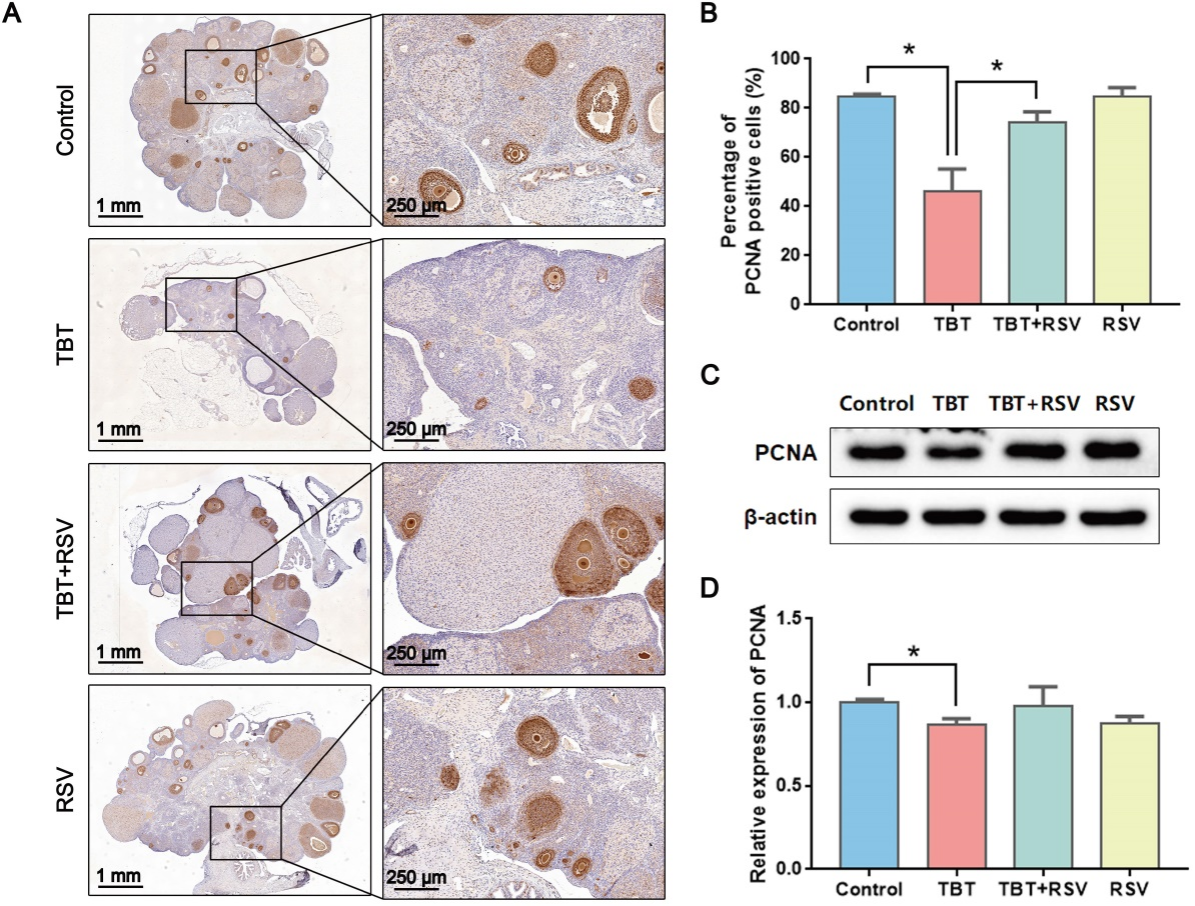
** Effects of RSV on PCNA expression in the ovaries of TBT-exposed rats. A,** Immunohistochemical analysis of PCNA in rat ovaries from the control group, the TBT group, the TBT + RSV group and the RSV group. **B,** The number of PCNA-positive GCs was counted in randomly selected fields. **C,** Western blotting analysis of PCNA in ovaries. **D,** PCNA protein expression was analyzed statistically. Data are presented as the mean ± SEM (n = 3-6, *p ≤ 0.05).

**Figure 4:**
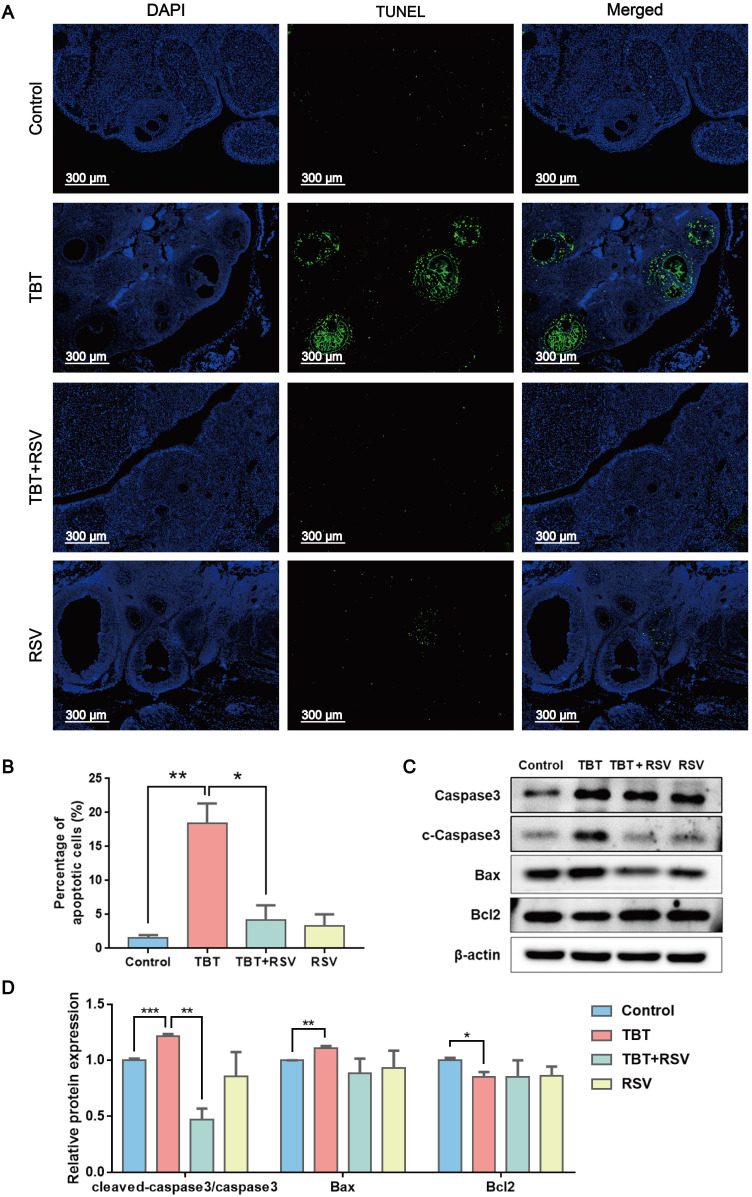
** RSV inhibited TBT-induced granulosa cell apoptosis. A,** TUNEL staining of rat ovaries treated with TBT and RSV. **B,** The number of TUNEL-positive GCs was counted in randomly selected fields. **C-D,** Protein expression of apoptosis-related genes detected by Western blot. Data are presented as the mean ± SEM (n = 3-6). Data were compared between two groups with Student's t-test, *p ≤ 0.05, **p ≤ 0.01, ***p ≤ 0.001.

GC proliferation and apoptosis predicts the fate of a follicle, and researchers have suggested that GC apoptosis is the basic mechanism of follicular atresia 30. To evaluate the status of follicular development, we detected the expression of proliferating cell nuclear antigen (PCNA), a typical marker of proliferation, in ovarian granulosa cells by immunohistochemistry. In the ovaries of the control rats, brown signals from PCNA-positive follicular GCs, including mural GCs and cumulus GCs, were detected. In TBT-treated rats, the number of positive cells decreased. After RSV treatment, PCNA expression in GCs was restored (Fig. 3A, B), which was further confirmed by Western blots (Fig. 3C, D). A terminal deoxynucleotidyl transferase dUTP nick end labeling (TUNEL) assay was performed to detect GC apoptosis in rat ovaries. TUNEL-positive signals were observed in the GCs, and TBT exposure resulted in significant increases in the percentage of apoptotic cells compared with those in the three other groups (Fig. 4A, B). The expression levels of apoptosis-related proteins were examined in ovarian tissues, revealing significant increases in both cleaved caspase-3/caspase-3 and Bax and a decrease in Bcl-2 in the TBT-exposed group compared with the control group. Additionally, RSV treatment recovered the expression of these proteins (Fig. 4C, D). Thus, these results suggested that RSV restored TBT-induced GC apoptosis to maintain folliculogenesis.

### RSV might alleviate TBT-induced cell projection assembly defects according to transcriptome analysis

**Figure 5:**
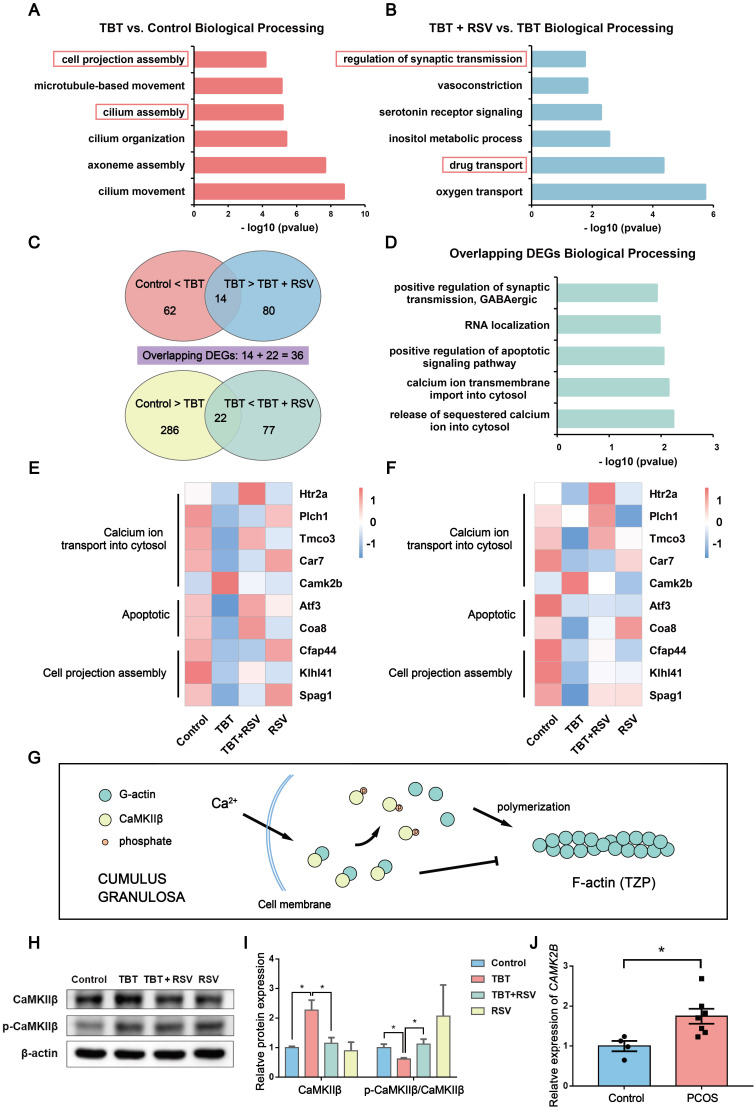
** RNA-seq analysis of ovarian tissue revealed overlapping gene expression linked to cell projection assembly and calcium ion transport. A,** Functional annotation charts derived using the Cluster Profiler R package for the differentially regulated genes in the TBT group versus the control group. Significance is indicated by the -log10 *p* value. **B,** Functional annotation charts derived using the Cluster Profiler R package for the differentially regulated genes in the TBT + RSV group versus the TBT group. Significance is indicated by the -log10 *p* value. **C,** Venn diagram of the 62 upregulated genes in the TBT rats compared to the control rats and the 80 downregulated genes in the TBT + RSV-treated rats compared to the TBT rats with 14 overlapping genes. Similarly, 22 overlapping genes were downregulated in TBT rats compared to the control rats and then upregulated in TBT + RSV-treated rats. **D,** Biological process GO term enrichment of the 36 overlapping DEGs. **E-F,** Heatmap showing the expression patterns of the overlapping DEGs and the confirmatory qPCR results. **G,** CaMKIIβ sequesters monomeric actin to inhibit actin polymerization, and Ca^2+^ activates calmodulin, which triggers CaMKIIβ dissociation from G-actins. **H,** Protein expression of CaMKIIβ and phospho-CaMKIIβ (Thr286). **I,** Analysis of the protein expression of CaMKIIβ and p-CaMKIIβ/CaMKIIβ. Data are presented as the mean ± SEM (n = 3-6). **J,** *CAMK2B* expression in COCs from women with PCOS and control women. Data were compared between two groups using Student's t-test, *p ≤ 0.05, **p ≤ 0.01, ***p ≤ 0.001.

To investigate the molecular mechanisms underlying “follicular dysplasia” in PCOS, we performed RNA sequencing (RNA-seq) analysis of ovaries dissected from 3 adult rats in estrus from each group and performed bioinformatics analysis. We performed a gene enrichment analysis with the differentially expressed genes (DEGs) by Gene Ontology (GO) enrichment (*p* ≤ 0.05, |log_2_ (fold change)| > 1; Fig. 5A, B). DEGs associated with biological processes in the TBT-exposed rats versus the control rats were mainly involved in cilium assembly and cell projection assembly (Fig. 5A). Compared to DEGs in the TBT-exposed rats, the differentially regulated genes in the TBT + RSV-treated rats were involved in drug transport and regulation of synaptic transmission (Fig. 5B).

To further understand how RSV treatment ameliorates TBT-induced follicular dysplasia, we identified 62 upregulated genes in TBT rats compared to control rats and 80 downregulated genes in TBT + RSV-treated rats compared to TBT rats with 14 overlapping genes. Similarly, 22 overlapping genes were downregulated in the TBT rats compared to the control rats and then upregulated in the TBT + RSV-treated rats (Fig. 5C). DEGs are listed in Table S1 and S2. Among the 36 overlapping DEGs, several were involved in calcium ion transmembrane import into the cytosol (5-hydroxytryptamine receptor 2A (*Htr2a*), phospholipase C, eta 1 (*Plch1*)), positive regulation of the apoptotic signaling pathway (activating transcription factor 3 (*Atf3*), cytochrome c oxidase assembly factor 8 (*Coa8*)) and RNA localization (Fig. 5D).

Next, heatmaps were constructed to show the expression patterns of the overlapping DEGs. *Htr2a*, *Plch1*, transmembrane and coiled-coil domains 3 (*Tmco3*) and carbonic anhydrase 7 (*Car7*), which are involved in calcium ion transmembrane import into the cytosol, were downregulated in the TBT rats but recovered in the TBT + RSV rats, whereas calcium-/calmodulin-dependent protein kinase II beta (*Camk2b*) was upregulated in the TBT rats and reduced in the TBT + RSV rats. The expression levels of *Atf3* and *Coa8* genes related to positive regulation of the apoptotic signaling pathway were decreased in the TBT group, and levels were recovered in the TBT + RSV group. Cilia- and flagella-associated protein 44 (*Cfap44*), Kelch-like family member 41 (*Klhl41*) and sperm-associated antigen 1 (*Spag1*), which are related to cell projection assembly, were downregulated in the TBT-treated rats, and expression levels were slightly improved by RSV (Fig. 5E). We then performed quantitative polymerase chain reaction (qPCR) to validate the DEGs presenting similar expression patterns for the genes selected from RNA sequencing (Fig. 5F).

Calcium/calmodulin-dependent protein kinase II beta (CaMKIIβ) is essential for the stability of the rigid filamentous actin system, which bundles actin filaments through a specific and stoichiometric interaction 31. However, CaMKIIβ can also sequester monomeric actin to inhibit actin polymerization 32. CaMKIIβ protein levels were significantly increased in the TBT-exposed rats compared with the controls and the TBT + RSV rats. Previous studies have shown that when the calcium concentration is increased, Ca^2+^ activates calmodulin, which triggers CaMKIIβ dissociation from actins 33 (Fig. 5G). Lower CaMKIIβ phosphorylation levels were also found in the TBT-exposed group, and these levels were restored upon RSV treatment (Fig. 5H, I).

To further investigate the pathogenesis of PCOS, we analyzed human PCOS samples from the clinic and the GEO database. *CAMK2B* mRNA expression was investigated in cumulus-oocyte complexes (COCs) sampled from follicles of women with and without PCOS, and the results showed that COCs from women with PCOS exhibited a significant increase in *CAMK2B* levels compared with healthy women (Fig. 5J, Table S4). Furthermore, the gene expression profile GSE155489 consists of 4 GC samples each from PCOS and healthy women. According to the criteria of a *p* value ≤ 0.05 and |log_2_ (fold change)| > 1, 1772 DEGs were identified between normal and PCOS GCs and were mostly involved in integral components of the plasma membrane and neuronal cell body according to GO enrichment analysis. *CAMK2B* expression was increased in PCOS GCs (*p* = 0.079; Fig. S3A, B). GSE168404, which contains 5 PCOS and 5 normal GC samples, was processed in the same manner. DEGs were mainly involved in cell-cell signaling and calcium ion binding, and *CAMK2B* expression levels were also increased in the PCOS group (*p* = 0.345; Fig. S3C, D). Taken together, given the changes in genes associated with microfilament assembly and the increase in CaMKIIβ in both ovaries from TBT-induced PCOS rats and GCs from PCOS patients, we considered that RSV might ameliorate follicular dysplasia via CaMKIIβ activation and cell projection assembly.

### RSV repaired TBT-induced oocyte-granulosa cell communication injury

The zona pellucida, a proteinaceous matrix that separates oocytes from the surrounding somatic cells termed GCs, is characterized by growing oocytes 34. GCs maintain contact with oocytes using TZPs rich in F-actin 35. Oocytes lack glycolysis and cholesterol metabolism. Therefore, oocyte growth and development rely on metabolites produced by GCs and transferred through TZPs to oocytes.

**Figure 6:**
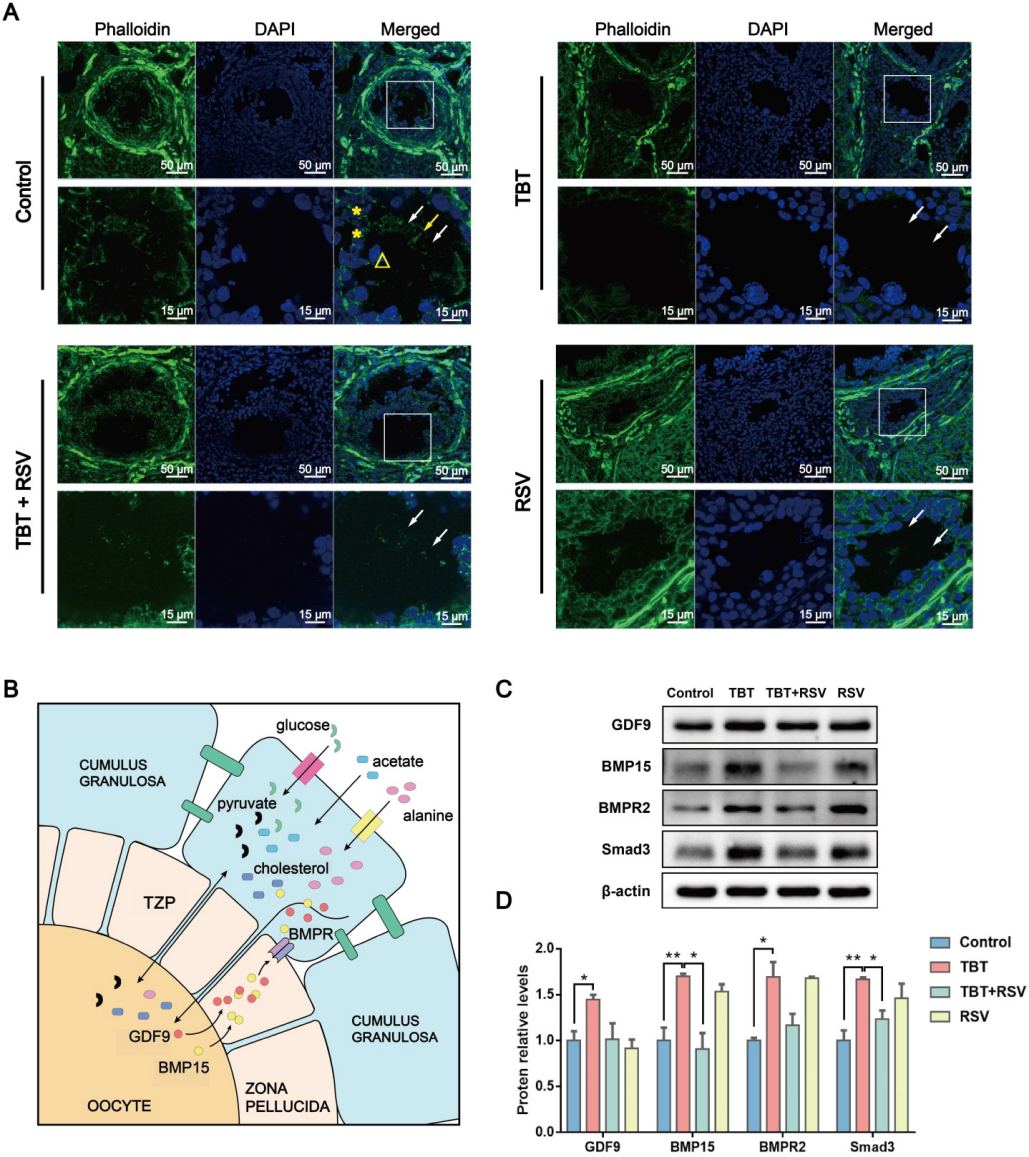
** RSV repaired TBT-induced oocyte-granulosa cell communication injury. A,** Phalloidin staining (green) of TZPs (arrow) formed from filamentous actin between oocytes (triangles) and GCs (asterisks). **B,** Oocyte-GC communication during growth. **C,** Protein expression of GDF9, BMP15, BMPR2 and Smad3. **D,** The results are expressed as the fold change in the optical density of a target protein, and β-actin expression served as the control. The mean protein expression of the control is designated as 1 in the graph. Data are presented as the mean ± SEM (n = 3-6). Data were compared between two groups with Student's t-test, *p ≤ 0.05, **p ≤ 0.01, ***p ≤ 0.001.

In this study, TZPs mainly consisting of F-actin were observed in frozen sections of ovaries. Confocal microscopy revealed TZPs labeled with Alexa Fluor 488 phalloidin that were clearly visible as thin filaments through the zona pellucida connecting GCs and oocytes in control rats. TZPs were minimally observed in the TBT-exposed group, yet an increase in the number of TZPs was observed in the TBT + RSV-treated group compared to the TBT group (Fig. 6A), indicating that RSV partly repaired the TZPs.

Oocyte-derived paracrine factors, mainly growth-differentiation factor 9 (GDF9) and bone morphogenetic protein 15 (BMP15), promote GC proliferation and differentiation 34. A study showed that GDF9 can promote TZP generation via Smad signaling 36. Impaired bidirectional communication between oocytes and GCs results in reduced oocyte competence in patients with PCOS 37. The levels of GDF9 and BMP15 protein secreted by oocytes were significantly increased in TBT-exposed rats, which might represent a consequence of negative feedback regulation. GDF9 and BMP15 released from oocytes can bind to their receptors on GCs and the bone morphogenetic protein type II receptor (BMPR2) gene, which then activates Smad3 signaling 38 (Fig. 6B). Here, we found that TBT significantly increased the levels of BMPR2 and Smad3. After RSV treatment, the protein levels were decreased to the same levels as those noted in the control group (Fig. 6C, D). Overall, our data showed that RSV ameliorated oocyte-GC bidirectional communication by restoring TBT-damaged TZPs.

## Discussion

PCOS is the most common endocrine disease in women of childbearing age 1, and studies have suggested that environmental and genetic mechanisms play an important role in the etiology of PCOS 39. In this study, we found for the first time that TZP damage plays an important role in the etiopathogenesis of PCOS. Moreover, a natural polyphenolic phytoalexin, RSV, ameliorates TBT-induced PCOS by recovering TZPs. Mechanistically, RSV allows calcium ion transport into the cytosol and activates CaMKIIβ to sustain the synthesis of TZPs. Notably, TZP damage and CaMKIIβ upregulation also occurred in human PCOS samples. In short, we demonstrated that RSV ameliorates TBT-induced PCOS via oocyte-GC communication.

In this experimental research, we studied PCOS induced by environmentally relevant levels of EDC instead of animal models of PCOS, which may simulate the incidence of this complicated endocrine syndrome in the population. We found that environmentally relevant levels of TBT inhibited the polymerization of microfilaments termed TZPs in ovaries and destroyed bidirectional oocyte-GC communication. A study found that focal adhesion kinase 2 (PTK2)-null oocytes exhibited a reduction in the number of TZPs and impaired dye coupling between oocytes and GCs 40. Researchers discovered that FSH increased the density of actin-TZPs physically linking GCs and oocytes and increased granulosa-oocyte gap junctional communication 41. Fullerenol nanoparticles accelerate TZP retraction from oocytes, disrupting the program of oocyte maturation and ultimately reducing oocyte quality 42. Injury to cumulus-oocyte communication has been proposed to play an important role in the process of PCOS 37, 43. Studies have shown that communication channels between GCs and oocytes in cumulus cell-oocyte complexes (COCs) from patients with PCOS are very weak compared with those of COCs from healthy women 44, 45. In our study, ovaries from TBT-exposed rats showed impaired TZPs and consequently abnormal oocyte-GC bidirectional communication. Moreover, we analyzed two independent GEO datasets and found abnormal synaptic transmission in GCs in PCOS 46, 47, which is consistent with the rat model induced by TBT. A recent study using a prenatal AMH-induced PCOS mouse model also showed that genes involved in nervous system development and axon guidance were hypermethylated in F3 ovaries 47. In this study, we discovered for the first time that TZP damage contributes to the pathological process of PCOS and may represent a target for PCOS therapy.

PCOS is associated with many complications, such as obesity 48, 49, insulin resistance 50, 51, and hyperandrogenism 52. However, in this study, TBT-induced PCOS rats did not show obvious obesity, insulin resistance (IR) or high levels of androgens. Nevertheless, TBT-induced PCOS rats showed disordered estrus cycles and termination of follicular development. Given that GnRH and FSH levels were not obviously altered in TBT-induced PCOS rats although estradiol and AMH levels were significantly altered, we focused on ovarian injury instead of disorder of the hypothalamic-pituitary-gonadal axis. A decrease in aromatase activity and consequently decreased estrogen levels were observed in PCOS 53. Estrogen also coordinates a series of gonadotropin secretions via a complex feedback loop and finally influences folliculogenesis 54, 55. In summary, these studies suggest that in contrast to the pathogenic mechanisms of hormone-induced PCOS, TBT acts directly on the process of follicular development during PCOS pathogenesis.

To investigate how TBT injures TZPs in ovarian follicles, we analyzed ovarian tissues using RNA-seq. We found that TZPs mediating oocyte-GC communication were severely damaged by CaMKIIβ upregulation in TBT-exposed rats. We also found an increase in *CAMK2B* expression in PCOS patients based on GEO database analysis. Less calcium ion transmembrane import into the cytosol also contributes to CaMKIIβ accumulation 56. Calcium channels are essential for neurotransmission, gene expression, and other physiological responses 57, and abnormal expression of channel proteins has been confirmed to be closely related to many clinical diseases 58. A recent study discovered that CaMKII activation protects retinal ganglion cells from injuries 59. In the TBT-exposed rats in our study, calcium ion-binding proteins in the plasma membrane were downregulated. RSV has been reported to activate Ca^2+^/calmodulin upon Ca^2+^ influx from the extracellular medium 60. In our study, RSV treatment improved the expression of calcium channel proteins. Therefore, we tentatively proposed that RSV increased the calcium concentration in the cytosol, activated CaMKIIβ, and released free actin monomers to synthesize TZPs.

In conclusion, we discovered a novel pathogenesis mechanism of PCOS mediated by TZP damage. Excessive CaMKIIβ sequesters monomeric actin to inhibit actin polymerization, resulting in failed TZP synthesis and damaged bidirectional communication between oocytes and granulosa cells in TBT-exposed rats. Moreover, RSV ameliorates ovarian failure and recovers estrus cycles in TBT-induced PCOS rats. Our study proposes the following underlying mechanism: RSV decreases CaMKIIβ expression, and RSV promotes the influx of calcium ions to activate CaMKIIβ phosphorylation. RSV restored TZP synthesis to maintain oocyte-GC communication in ovarian follicles, thus alleviating PCOS symptoms. Our study offers a new perspective on the pathogenic mechanism of PCOS and demonstrates that RSV represents a potential natural remedy for the treatment of PCOS, which is meaningful for improving women's health and reproduction.

## Materials and methods

### PCOS patients' samples

All patients received *in vitro* fertilization (IVF) treatment between July 1, 2021 and August 30, 2021 in Xiamen University Affiliated Chenggong Hospital. All PCOS patients met the Rotterdam criteria for diagnosis 61. The inclusion criteria for nonPCOS controls were (1) age between 20 and 35 years; (2) regular menstrual cycles ranging from 25 to 35 days; (3) body mass index (BMI) ≤ 28 kg/m^2^; and (3) normal basal serum FSH (≤ 10 mIU/mL) and estradiol (E2) (≤ 75 pg/mL). Exclusion criteria for controls were any diagnosis of chronic conditions, metabolism-related disorders, diminished ovarian reserve, and endometriosis. Institutional review board approval was obtained from the Ethical Committee of Medical College Xiamen University. All the subjects enrolled in this study provided written formal consent before participation.

During the ovum-pick up procedure of the treatment, GCs were dissected from the COCs under a stereoscope (Olympus, Japan) with glass Pasteur pipettes and separated from follicular fluid with centrifugation at 2000 rpm for 10 min. The GCs were then lysed in 1 mL TRIzol reagent (Takara, China) for subsequent analyses.

### Animal care and treatment

All treatments and procedures involving animals were performed in accordance with the animal ethical principles adopted by the Animal Experimental Center of Xiamen University (Approval No: XMULAC20170361). Sprague-Dawley rats weighing 200-250 g were obtained from SLAC Laboratory Animal Co., Ltd. (Shanghai, China) and were maintained in a pathogen-free environment with a constant temperature (24 ± 1 °C) and a 12-h light-dark cycle. After more than one week of acclimatization, rats were mated at a 2:1 female-male ratio. Pregnant rats were housed in individual cages once pregnancy was detected. The pups in each litter were randomly reduced to five females on postnatal day 1 (PND1) for the following experiment. The female pups were divided into four groups: the control group (n = 13), the TBT group (n = 18), the TBT + RSV group (n = 14) and the RSV group (n = 12). Pups from one litter were allocated to the same group to avoid cross contamination.

TBT and RSV at a purity of 99% were purchased from Sigma-Aldrich (USA). Rats in the control group and the RSV group were treated daily with vehicle (10% ethanol with 90% olive oil), and rats in the TBT group and the TBT + RSV group were treated daily with 100 ng/kg/d TBT in olive oil by subcutaneous injection from PND 1 to 21 (1 μL g^-1^ body weight). The pups were separated from their mothers. After 3 days of acclimatization, the TBT + RSV group and the RSV group rats were administered RSV orally at 20 mg/kg/d in 0.5% sodium carboxymethylcellulose (CMC), and the control group and the TBT group rats were administered 0.5% CMC for 28 days.

### Estrus cycle analysis

Vaginal smears were collected from 2-month-old adult rats at approximately 9 am every day for 20 consecutive days. Estrus cycle stages were visually examined by cytology under a microscope 17.

### Blood and tissue collection

After 21 days of TBT exposure and 28 days of RSV treatment, rat blood was collected from the submandibular vein. At the end of the experiment, the animals were anesthetized by intraperitoneal injection of 7% chloral hydrate (0.5 mL/100 g) in the estrus phase. Blood was collected by cardiac puncture and centrifuged at 2500 rpm for 20 min at 4 °C. E2, T, P, LH, FSH, GnRH and AMH levels in blood serum were assayed using commercial radioimmunoassay kits by Beijing Sino-UK Institute of Biological Technology (Beijing, China). Ovaries were harvested for subsequent studies.

### Total tin analysis

Dried liver samples were digested with 1 mL purified 65% HNO_3_ (Merck, Germany) at 80 °C for at least 4 hours. The digests were then diluted to 10 mL with Milli-Q water. The total tin concentrations in liver samples were determined by measuring tin isotope ^118^Sn using inductively coupled plasma mass spectrometry (PerkinElmer NexION 2000). The tin standard (GSB 04-1753-2004) was diluted with 2% HNO_3_. The R^2^ value of the calibration curve was 0.999.

### Histology and immunostaining

Ovarian tissues were fixed in 4% paraformaldehyde (PFA), embedded in paraffin, and sliced into 5-µm sections. Every fifth slide was stained with hematoxylin and eosin (H&E) for differential follicle counts. The corpus luteum, cysts, antral follicles and atretic follicles were defined according to a previous study 62.

Sample sections were processed for immunohistochemistry to assess GC proliferation. The sections were incubated with PCNA antibody (Abcam, Britain) overnight at 4 °C. The next day, the sections were incubated with an anti-mouse IgG-HRP-linked antibody (Sigma-Aldrich, USA) for 60 minutes at room temperature. Subsequently, the sections were visualized with a diaminobenzidine tetrahydrochloride (DAB) substrate chromogen system (Boster, China) at room temperature for 3 minutes. Negative controls were incubated with phosphate-buffered saline (PBS) instead of the PCNA antibody and showed no staining. The proliferation index of PCNA-positive cells was determined by the average number of positively stained GCs divided by the average total number of GCs in the area multiplied by 100.

### TUNEL assay

Apoptosis induction was analyzed using the DeadEnd Fluorometric TUNEL System (Promega, USA). According to the manufacturer's instructions, each section was incubated with TUNEL reaction mixture at 37 °C for 1 h. The green fluorescence of the apoptotic GCs was examined using Versa 200 (Leica, Germany). The number of TUNEL-positive GCs was calculated using Image-Pro Plus.

### Western blotting

Total protein isolated from rat ovaries was used for Western blotting. Extracts containing equivalent quantities of proteins were separated by sodium dodecyl sulfate-polyacrylamide gel electrophoresis (SDS-PAGE, 7.5-12% gels) and transferred onto polyvinylidene fluoride (PVDF) membranes (Millipore, USA). The antibodies used included β-actin (Sigma-Aldrich, USA); PI3K, Akt, mTOR, PCNA, cleaved-caspase3, Bax, Bcl2, and phospho-CaMKII (Abcam, Britain); phospho-Akt, GDF9, BMP15, and BMPR2 (Abclonal, China); Smad3 (Beyotime, China); caspase3 (Bioss, China) and CaMKII (Cell Signaling Technology, USA). The protein band corresponding to protein specifically bound by the primary antibody was detected by an anti-rabbit or an anti-mouse IgG-HRP-linked antibody (Sigma-Aldrich, USA). Antibody-reactive bands were visualized using chemiluminescence (Sigma-Aldrich, USA).

### RNA extraction and mRNA expression analysis

Ovaries were homogenized in 1 mL TRIzol reagent (Takara, China) with a tissue homogenizer, and total RNA was isolated following the manufacturer's instructions. cDNA was synthesized using the TransScript First-Strand cDNA Synthesis SuperMix Kit (Transgen, China). Quantitative real-time PCR was performed on a Stratagene Mx3000 P real-time PCR system (Stratagene, USA) with a mixture of TransStart Tip Green qPCR SuperMix (Transgen, China).

The primer sequences are shown in Table S3. *Gapdh* (glyceraldehyde-3-phosphate dehydrogenase) served as the endogenous control, and the relative expression levels of mRNA are presented as 2^-ΔΔCt^ values.

### RNA sequencing and analysis

Total RNA was extracted from rat ovaries using TRIzol reagent (Takara, China) for RNA-seq experiments (three biological replicates for each group). RNA-seq was performed using Illumina Sequencing (Novogene, China). Raw reads were mapped to the rat reference genome (mRatBN6.0) using STAR default mapping parameters. DEGs between two groups were identified by a log_2_ (fold change) < - 1 or > 1 and a *p* value ≤ 0.05. GO analysis of DEGs was implemented using the clusterProfiler R package.

### Phalloidin staining

Tissue sections were procured by embedding rat ovaries in optical cutting temperature compound (Sakura, USA). The ovaries were immediately frozen in liquid nitrogen and cut into 10-μm cryosections using a cryostat (CM 1950, Leica, USA). Cryosections were fixed in 4% formaldehyde for 10 minutes and washed thrice in PBS containing 0.1% Triton X-100 for 5 minutes. Sections were blocked with 1% bovine serum albumin (YHSM, China) at room temperature for 1 hour. The sections were then incubated with Alexa Fluor 488 phalloidin (Thermo Fisher, USA) at 37 °C for 30 minutes and 4'-6-diamidino-2-phenylindole (DAPI) (Sigma-Aldrich, USA) for 3 minutes. Images were acquired using Zen Blue 3.1 and LSM 900+ Airyscan2 confocal microscopes (Zeiss, Germany).

### Statistical analysis

Data were expressed as the means ± standard error of the mean (SEM). Comparisons between two groups were analyzed using unpaired Student's t-test and IBM SPSS Statistics 19 (IBM, USA). Statistical analyses were performed with the GraphPad Prism 7 program, and *p* ≤ 0.05 was considered statistically significant (represented as ^*^p ≤ 0.05, ^**^p ≤ 0.01, ^***^p ≤ 0.001).

## Supplementary Material

Supplementary figures and tables.

## Abbreviations

PCOS: Polycystic ovary syndrome
GC: granulosa cell
TZP: transzonal projection
EDC: environmental endocrine disruptor
TBT: tributyltin
RSV: resveratrol
GEO: gene expression omnibus
P: progesterone
LH: luteinizing hormone
FSH: follicle-stimulating hormone
T: testosterone
GnRH: gonadotropin-releasing hormone
E2: estradiol
AMH: anti-Müllerian hormone
TGF-β: transforming growth factor beta
PI3K: phosphatidylinositol 3-kinase
Akt: protein kinase B
mTOR: mammalian target of rapamycin
TUNEL: terminal deoxynucleotidyl transferase dUTP nick end labeling
RNA-seq: RNA sequencing
DEG: differentially expressed gene
CaMKIIβ: calcium/calmodulin-dependent protein kinase II beta
GO: gene ontology
GDF9: growth-differentiation factor 9
BMP15: bone morphogenetic protein 15
BMPR2: bone morphogenetic protein type II receptor
PND: postnatal day
