# Supplementary material for: Resveratrol ameliorates polycystic ovary syndrome via transzonal projections within oocyte-granulosa cell communication
Source: Theranostics. 2022 Jan 1;12(2):782–95. doi: 10.7150/thno.67167 (PMC8692920; doi:10.7150/thno.67167)
Supplement: Supplementary file 1 — Supplementary figures and tables. [file thnov12p0782s1.pdf]

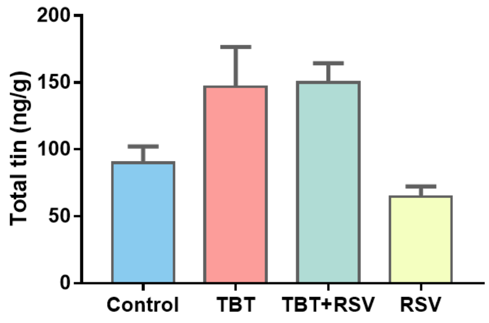

Figure S1. The total tin concentrations in livers.

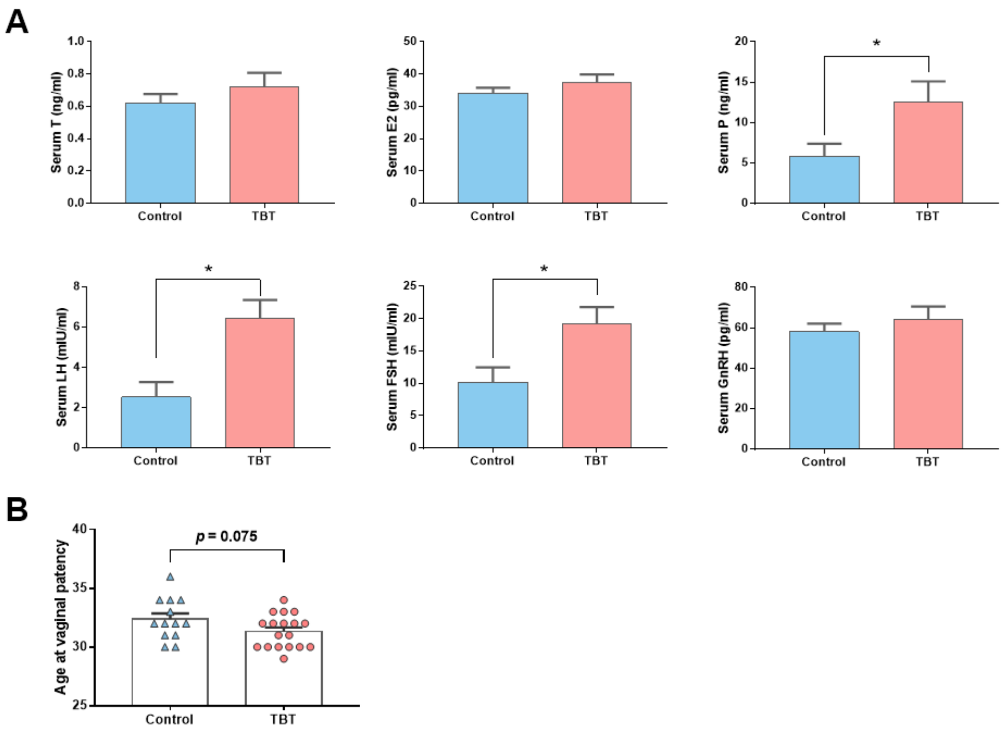

Figure S2. Effects of TBT exposure on the early development of infant rats. A, The hormone levels of infant rats (PND21) after TBT exposure. **B,** Sexual maturation (vaginal patency) of the rats in the control group and TBT + RSV group. Data are presented as the mean ± SEM. Data were compared between two groups with Student's t-test, $^{*}p \leq 0.05$

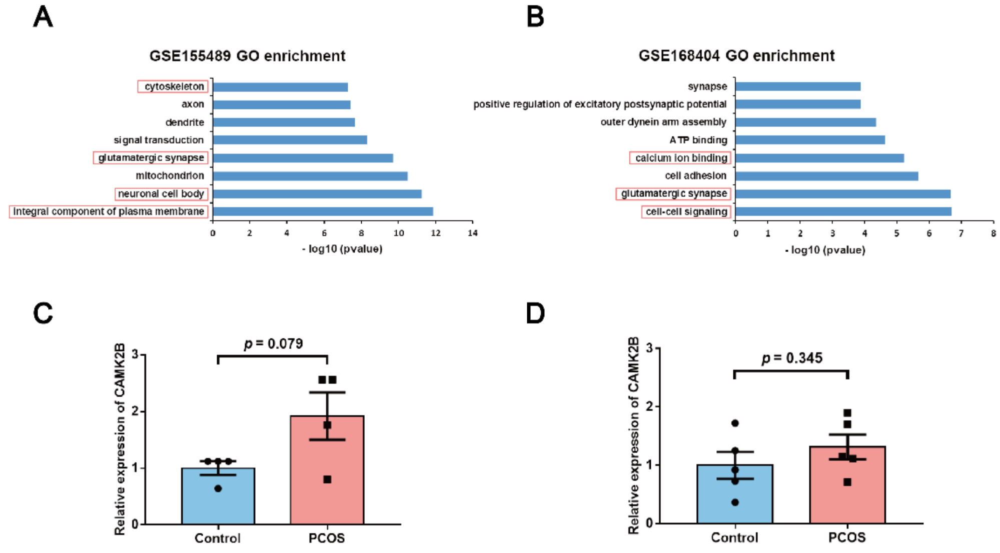

Figure S3. GO enrichment in PCOS patients from GEO database. A-B, Gene ontology (GO) enrichment analysis and *CAMK2B* expression of GSE155489 (n = 4). **C-D,** Gene ontology (GO) enrichment analysis and *CAMK2B* expression of GSE168404 (n = 5). Data are presented as the mean ± SEM. Data were compared between two groups with Student's t-test, $^{*}p \leq 0.05$

Table S1. Differentially expressed genes in ovaries from TBT-treated rats compared to control rats. This table lists all genes detected on differential expression analysis from RNA-seq experiments at a cutoff of $\log_2$ (fold change) < - 1 or > 1 and q ≤ 0.05.

Gene name	Gene ID	log2FoldChange	*P* value
Atp5pb	ENSRNOG00000016000	-4.4884	3.00E-09
Nr1i2	ENSRNOG00000002906	-4.24667	3.25E-07
Maats1	ENSRNOG00000002976	-4.70186	4.26E-06
Nxpe5	ENSRNOG00000031343	-5.12893	2.43E-05
LOC100910882	ENSRNOG00000049814	2.883724	9.83E-05
LOC103693999	ENSRNOG00000019346	-3.08177	0.000288

Kansl2	ENSRNOG00000053541	-2.9783	0.000374
LOC688553	ENSRNOG00000037632	-6.60179	0.000397
Olr1588	ENSRNOG00000003509	5.883607	0.000474
Rbm39	ENSRNOG00000019848	-1.87992	0.000503
Ank3	ENSRNOG00000053288	-2.51516	0.000606
LOC108348062	ENSRNOG00000040153	-2.39434	0.000675
Gbp6	ENSRNOG00000002134	-1.48706	0.000787
Ugt8	ENSRNOG00000009345	-7.06181	0.000923
Ctsw	ENSRNOG00000027096	1.879523	0.001092
Slc16a14	ENSRNOG00000017072	-2.9249	0.001095
Atn1	ENSRNOG00000049575	1.884897	0.001096
Oas1a	ENSRNOG00000001369	-1.60554	0.001128
LOC108348050	ENSRNOG00000050713	-4.56107	0.001196
Cttn	ENSRNOG00000047280	-1.60916	0.001318
Matn1	ENSRNOG00000010932	-4.90777	0.001431
Grik2	ENSRNOG00000000368	-3.28955	0.001664
Ehf	ENSRNOG00000007484	-3.54521	0.001714
Srm	ENSRNOG00000046379	3.277183	0.001738
Gjb1	ENSRNOG00000003746	-6.83064	0.001765
Sfrp4	ENSRNOG00000054957	-1.67437	0.001862
Ttll9	ENSRNOG00000008596	-1.84088	0.002088
Ap3b2	ENSRNOG00000019249	-3.0617	0.002148
Tmprss2	ENSRNOG00000001976	-3.284	0.002164
LOC103690026	ENSRNOG00000062153	4.279407	0.002358
Cfap44	ENSRNOG00000028077	-3.62409	0.002387
Ccdc113	ENSRNOG00000051510	-3.76422	0.002401
Prxl2a	ENSRNOG00000011140	-2.32453	0.002436
Scn9a	ENSRNOG00000006639	-3.58451	0.00262
Plch1	ENSRNOG00000009955	-3.49061	0.003033
LOC108348114	ENSRNOG00000047573	1.573307	0.003302
Herc6	ENSRNOG00000023969	-1.09698	0.003312
Car7	ENSRNOG00000012371	-4.48431	0.003326
AC098547.1	ENSRNOG00000045877	1.604622	0.003482
LOC501233	ENSRNOG00000015438	-2.30628	0.00356
Ush2a	ENSRNOG00000003738	-4.24973	0.003933
Spaca9	ENSRNOG00000012697	-5.01359	0.003938
Lrrc43	ENSRNOG00000008470	-4.35743	0.004377
Spata18	ENSRNOG00000002120	-3.11049	0.004735
Il1rl1	ENSRNOG00000014835	2.749836	0.005353
Cfap221	ENSRNOG00000056781	-5.26762	0.005398
Kif6	ENSRNOG00000011453	-6.82842	0.00546
Lrriq4	ENSRNOG00000009004	-4.60141	0.005674
Nox4	ENSRNOG00000013925	-1.83451	0.005907
AABR07005983.1	ENSRNOG00000005953	-6.24985	0.00607

Lman1l	ENSRNOG00000019355	-6.28417	0.00624
Cd3g	ENSRNOG00000015945	1.928049	0.006275
Hydin	ENSRNOG00000017178	-2.33253	0.006285
Cd209	ENSRNOG00000001017	5.806585	0.006301
LOC100912578	ENSRNOG00000059883	-1.88278	0.006744
Ecrg4	ENSRNOG00000023576	-1.60415	0.006967
Fgf1	ENSRNOG00000013867	-2.04985	0.007032
Crb3	ENSRNOG00000047322	-2.59747	0.00706
Wnt9b	ENSRNOG00000003807	3.238134	0.007062
Gal	ENSRNOG00000015156	1.850512	0.007503
Prr18	ENSRNOG00000024149	-6.88754	0.007564
Cpne5	ENSRNOG00000000522	-2.21646	0.008561
Dok6	ENSRNOG00000038190	3.737471	0.008674
LOC100911881	ENSRNOG00000050181	2.166258	0.008776
LOC100912262	ENSRNOG00000050193	-2.7115	0.009241
Nectin4	ENSRNOG00000004029	-2.99657	0.009322
Klk1	ENSRNOG00000032857	-3.07854	0.009388
Slc13a2	ENSRNOG00000010337	2.005309	0.009501
AC097129.1	ENSRNOG00000031408	-2.67569	0.009643
Ttll6	ENSRNOG00000004939	-1.43667	0.009725
RGD1309139	ENSRNOG00000020307	-4.05598	0.009999
Ddo	ENSRNOG00000000582	-3.92425	0.010075
Rlim	ENSRNOG00000002824	-3.26202	0.01027
Hnrnpab	ENSRNOG00000003645	1.641013	0.010343
Dnah5	ENSRNOG00000048363	-6.67962	0.010421
Pomp	ENSRNOG00000049229	1.156559	0.010453
Mthfd1l	ENSRNOG00000019582	1.098548	0.010464
Spag1	ENSRNOG00000010078	-1.10639	0.010497
LOC103690317	ENSRNOG00000060185	3.793487	0.010506
Cndp1	ENSRNOG00000027739	-2.46963	0.010661
Cfap45	ENSRNOG00000008492	-2.32152	0.011106
Armc4	ENSRNOG00000018905	-4.74268	0.011384
AABR07013464.1	ENSRNOG00000023965	-3.45903	0.011542
AABR07066693.1	ENSRNOG00000047003	-2.74403	0.01156
Insc	ENSRNOG00000024712	-2.88019	0.011609
Klk1c3	ENSRNOG00000033414	-5.49858	0.011666
Creb3l4	ENSRNOG00000023493	-2.92459	0.01172
Pgc	ENSRNOG00000014492	-5.10649	0.011804
Plppr4	ENSRNOG00000016872	-1.65616	0.011902
Sgsm1	ENSRNOG00000000708	-2.34894	0.011997
Junb	ENSRNOG00000042838	-1.37543	0.012261
Mmp13	ENSRNOG00000008478	-1.84167	0.01257
Lrrc34	ENSRNOG00000027935	-2.64533	0.012694
Ak7	ENSRNOG00000055714	-3.05281	0.012719

Cd163	ENSRNOG00000010253	1.463176	0.012747
Chac1	ENSRNOG00000014387	-2.40168	0.013224
Trpv5	ENSRNOG00000015394	-5.61542	0.013409
LOC108348065	ENSRNOG00000047281	-1.25432	0.013459
AC126641.1	ENSRNOG00000011160	-2.47121	0.013564
LOC681006	ENSRNOG00000025953	-6.49863	0.013676
Loxl4	ENSRNOG00000015727	-1.12345	0.013687
Smpd3	ENSRNOG00000000257	-2.23295	0.013693
Rab17	ENSRNOG00000052979	-4.36266	0.013942
LOC688801	ENSRNOG00000042867	-5.68026	0.013954
Rragd	ENSRNOG00000007331	-1.36743	0.014013
AABR07029452.1	ENSRNOG00000058208	-1.72917	0.01424
Atf3	ENSRNOG00000003745	-3.51331	0.014335
Slc15a2	ENSRNOG00000002305	-2.26435	0.014669
Ncbp2	ENSRNOG00000001746	2.30411	0.014883
Fbp1	ENSRNOG00000017597	-3.92798	0.014906
Cfi	ENSRNOG00000053400	-3.20117	0.014963
Unc93a	ENSRNOG00000047027	-5.19702	0.015024
Oxt	ENSRNOG00000021225	-5.98135	0.015056
Klhl41	ENSRNOG00000007461	-2.32486	0.015163
LOC103690018	ENSRNOG00000023386	-2.77899	0.015195
AABR07042611.1	ENSRNOG00000045837	3.169921	0.015534
AC131483.1	ENSRNOG00000007816	-3.57635	0.015809
Pcsk6	ENSRNOG00000011526	1.594174	0.015823
Lefty1	ENSRNOG00000003263	-6.39884	0.015839
AABR07016141.1	ENSRNOG00000046536	-3.70821	0.016189
Dsp	ENSRNOG00000013928	-1.85211	0.016706
Bhlha15	ENSRNOG00000025164	-2.53928	0.016724
Cwh43	ENSRNOG00000002174	-4.81848	0.016728
Actg2	ENSRNOG00000029401	-1.35845	0.016825
Tmc5	ENSRNOG00000036760	-4.40259	0.017024
Cfap99	ENSRNOG00000058434	-2.59835	0.017054
Gimap7	ENSRNOG00000038740	1.22764	0.01716
Coa8	ENSRNOG00000011542	-2.67219	0.017248
AABR07054096.1	ENSRNOG00000011028	-3.24846	0.017308
Esrp1	ENSRNOG00000008184	-2.50086	0.017556
Ttll2	ENSRNOG00000033901	-7.09728	0.017556
Aldh1a3	ENSRNOG00000052070	-1.28439	0.017684
Hhip	ENSRNOG00000018268	2.280733	0.017894
RGD1564801	ENSRNOG00000048095	3.821499	0.018411
Surf2	ENSRNOG00000047187	-2.28951	0.018441
LOC100911660	ENSRNOG00000047692	2.7663	0.01863
Zbbx	ENSRNOG00000009792	-7.05419	0.018639
Cfap126	ENSRNOG00000034221	-2.32197	0.018641

Gdpd2	ENSRNOG00000002800	-3.02642	0.019166
Btnl10	ENSRNOG00000043019	1.678757	0.019174
Fam83e	ENSRNOG00000021039	-3.78824	0.019355
AABR07071198.1	ENSRNOG00000053034	-2.60776	0.019386
Dnajb4	ENSRNOG00000013011	-1.76937	0.019406
AABR07005597.1	ENSRNOG00000050426	-7.00977	0.019816
Rsph1	ENSRNOG00000057862	-2.94647	0.019848
AABR07044375.1	ENSRNOG00000050215	-2.37315	0.020065
Krtap13-1	ENSRNOG00000042531	6.224739	0.02025
Cldn2	ENSRNOG00000054495	-2.05096	0.020534
Spef2	ENSRNOG00000058275	-2.00487	0.020605
LOC108348155	ENSRNOG00000050981	2.463759	0.020774
AABR07042821.1	ENSRNOG00000054489	-1.07876	0.020866
AABR07013802.1	ENSRNOG00000058222	-1.94015	0.020958
Tbcb	ENSRNOG00000020781	-2.28068	0.020972
LOC100910130	ENSRNOG00000058439	-2.65554	0.021005
Pnliprp2	ENSRNOG00000017982	-2.77828	0.021244
Fam111a	ENSRNOG00000012067	-2.19579	0.021343
AABR07001780.1	ENSRNOG00000056729	-3.74317	0.021592
Mal2	ENSRNOG00000008615	-2.01237	0.021909
SNORD83	ENSRNOG00000055027	3.447611	0.021949
Fam3d	ENSRNOG00000007320	-5.08612	0.022108
Acta1	ENSRNOG00000017786	-2.31889	0.022477
Pvalb	ENSRNOG00000006471	-6.91662	0.022502
Rp1l1	ENSRNOG00000046326	-3.7753	0.022549
LOC680875	ENSRNOG00000023828	-3.92277	0.022766
Fam50a	ENSRNOG00000045790	-2.60766	0.022769
Fam189a2	ENSRNOG00000061147	-1.81635	0.022996
Calb2	ENSRNOG00000016977	-1.64945	0.02325
Cmahp	ENSRNOG00000003094	-2.80852	0.023452
Zgrf1	ENSRNOG00000036980	-1.17172	0.023481
Nfe2l3	ENSRNOG00000010886	-3.02967	0.023731
Ifit1	ENSRNOG00000019050	4.212692	0.023805
Emb	ENSRNOG00000060329	-1.65964	0.02391
Alox12e	ENSRNOG00000019074	-5.28896	0.023976
Ada	ENSRNOG00000010265	-1.74895	0.024135
Mx2	ENSRNOG00000001963	-1.09081	0.024282
AABR07024641.1	ENSRNOG00000055192	-6.09312	0.024485
Pik3ap1	ENSRNOG00000013309	-1.35664	0.02475
RGD1309350	ENSRNOG00000048275	-2.93864	0.024781
Cd164l2	ENSRNOG00000009505	-2.989	0.024883
Efhb	ENSRNOG00000012618	-6.02495	0.024922
Slc22a3	ENSRNOG00000022946	-2.04745	0.025051
Slc34a2	ENSRNOG00000004626	-2.9426	0.025079

Tbx20	ENSRNOG00000016181	-2.93371	0.025138
Hmox1	ENSRNOG00000014117	-3.32017	0.025385
Ppp2r2b	ENSRNOG00000018851	-2.29731	0.025549
LOC100911672	ENSRNOG00000060594	1.4038	0.026248
Pbx4	ENSRNOG00000010736	-1.09671	0.026252
Fgf16	ENSRNOG00000061530	-3.27616	0.026294
AABR07027088.1	ENSRNOG00000046068	2.625658	0.02642
B3gat2	ENSRNOG00000046852	-2.55875	0.026577
Cyp2ab1	ENSRNOG00000042367	1.113436	0.026677
Ankrd22	ENSRNOG00000019190	-5.32924	0.027341
Morc4	ENSRNOG00000060846	-1.7136	0.027376
Angptl3	ENSRNOG00000008638	-5.32513	0.027555
LOC680885	ENSRNOG00000039749	-6.76453	0.027575
Cbarp	ENSRNOG00000024349	-1.31708	0.028011
Fam216b	ENSRNOG00000021943	-5.93024	0.028106
P2rx7	ENSRNOG00000001296	-1.13744	0.028222
AABR07036374.1	ENSRNOG00000057023	4.645532	0.028256
Adgrd1	ENSRNOG00000023536	-2.78865	0.02837
Htr2a	ENSRNOG00000010063	-1.5098	0.028493
Hoxb4	ENSRNOG00000008191	-2.02908	0.028653
Ropn1l	ENSRNOG00000042781	-2.2847	0.028891
Hoxb3	ENSRNOG00000008313	-1.22401	0.028916
Kcnq2	ENSRNOG00000011624	-4.7308	0.029021
Kif9	ENSRNOG00000020891	-1.75242	0.029091
Rspo3	ENSRNOG00000011568	-3.86992	0.029092
Fam71a	ENSRNOG00000029371	-5.95942	0.029382
Pifo	ENSRNOG00000016842	-6.71005	0.02962
Galnt12	ENSRNOG00000008099	-1.78846	0.029668
Ccl5	ENSRNOG00000010906	1.282548	0.029829
Wdr66	ENSRNOG00000001342	-2.13428	0.030151
Cpxm1	ENSRNOG00000021220	-1.55791	0.030915
Trib3	ENSRNOG00000007319	-1.66997	0.031028
Sox9	ENSRNOG00000002607	-1.98106	0.031043
Rbm24	ENSRNOG00000046547	-2.92703	0.031355
Zfp474	ENSRNOG00000018724	-5.90661	0.031542
Rasl11b	ENSRNOG00000002097	-1.28376	0.031643
Ahr	ENSRNOG00000004342	-1.11575	0.031653
LOC103689951	ENSRNOG00000048049	-3.40812	0.032434
Rgs1	ENSRNOG00000003895	-1.25337	0.03273
Rbm12	ENSRNOG00000046990	1.261699	0.032776
Fam92b	ENSRNOG00000021714	-2.51973	0.0328
AABR07021591.1	ENSRNOG00000029459	-1.58477	0.032996
Ccl6	ENSRNOG00000030021	1.185088	0.033012
Rhpn2	ENSRNOG00000011885	-1.75672	0.033294

AC116220.1	ENSRNOG00000016854	-1.39382	0.033295
Myo7b	ENSRNOG00000015035	-2.25356	0.033449
AC125757.1	ENSRNOG00000028991	1.715809	0.033517
Hoxb7	ENSRNOG00000007611	-2.13708	0.033628
Tent5a	ENSRNOG00000010240	-1.13607	0.033869
LOC102554838	ENSRNOG00000031595	-5.7754	0.033959
LOC100911002	ENSRNOG00000050177	4.029392	0.033995
Mir324	ENSRNOG00000035519	3.928199	0.034013
Alox15b	ENSRNOG00000007778	-3.64235	0.034039
Hoxb2	ENSRNOG00000008365	-1.76619	0.034345
Sik1	ENSRNOG00000001189	-1.03456	0.034466
Lrcol1	ENSRNOG00000037466	-4.43484	0.034788
AABR07031521.1	ENSRNOG00000062247	-1.48897	0.034817
Enpp5	ENSRNOG00000010232	-1.0272	0.034834
Ehhadh	ENSRNOG00000001770	-1.2145	0.034956
Cnksr1	ENSRNOG00000022838	-1.83702	0.035338
Hspa8	ENSRNOG00000034066	-1.48839	0.035423
Acsm3	ENSRNOG00000032246	-1.83917	0.035472
Tent5c	ENSRNOG00000015222	-1.05725	0.035617
Hamp	ENSRNOG00000021029	-1.01822	0.035665
Armh1	ENSRNOG00000031494	-4.22754	0.035735
Saxo2	ENSRNOG00000026296	-2.87143	0.036066
Tspan8	ENSRNOG00000004411	-1.50053	0.036131
Glb1l2	ENSRNOG00000007561	-1.41157	0.03616
AABR07044375.2	ENSRNOG00000057822	-2.66389	0.036165
Pik3c2g	ENSRNOG00000034228	-2.62018	0.036203
AABR07000738.1	ENSRNOG00000052999	1.693831	0.036369
Dcdc2	ENSRNOG00000017511	-2.11984	0.036641
Eno2	ENSRNOG00000013141	2.414318	0.036842
Ap1m2	ENSRNOG00000043093	-2.93381	0.036944
Tmem52	ENSRNOG00000016618	-4.23126	0.036946
LOC688932	ENSRNOG00000030228	-1.92112	0.036987
Popdc3	ENSRNOG00000047102	-6.53307	0.037153
Gdf15	ENSRNOG00000019661	-1.63597	0.037236
Ppp1r35	ENSRNOG00000024505	2.040557	0.037316
Crhr1	ENSRNOG00000004900	-4.10304	0.037349
Zscan10	ENSRNOG00000021782	-3.06453	0.037673
Cxcl17	ENSRNOG00000037814	-2.04428	0.037696
RGD1562726	ENSRNOG00000028670	-2.74739	0.037789
Mir6334	ENSRNOG00000049688	-2.45564	0.037792
Dnah11	ENSRNOG00000005451	-1.45411	0.037821
Tjp3	ENSRNOG00000020501	-1.01158	0.037859
Snap91	ENSRNOG00000023861	1.308502	0.038076
LOC690276	ENSRNOG00000024338	-1.73537	0.038167

RNaseP_nuc	ENSRNOG00000059884	4.913604	0.03852
Hapln1	ENSRNOG00000032002	6.496733	0.038563
Shcbp1l	ENSRNOG00000002691	-4.8508	0.038634
Ttc34	ENSRNOG00000024526	-5.66209	0.038853
AABR07041949.1	ENSRNOG00000058173	-5.66209	0.038853
Scx	ENSRNOG00000021812	-1.04602	0.038917
Btk	ENSRNOG00000052407	-1.52132	0.039035
LOC100911486	ENSRNOG00000000156	1.349766	0.039058
Bhlhe40	ENSRNOG00000007152	-1.16855	0.039144
Tll1	ENSRNOG00000033528	2.036526	0.039294
Krt36	ENSRNOG00000031805	-4.17607	0.039893
Rsph14	ENSRNOG00000001315	-4.57319	0.039932
Clec4b2	ENSRNOG00000027655	4.290341	0.040118
Rnf39	ENSRNOG00000000781	-1.96278	0.040301
Tmco3	ENSRNOG00000046973	-2.45067	0.040446
Birc3	ENSRNOG00000005731	-1.39716	0.040792
Hnrnpc	ENSRNOG00000011621	1.131991	0.040987
AC109737.1	ENSRNOG00000014312	1.054693	0.041179
Trpv6	ENSRNOG00000014714	-1.54865	0.041426
Dnai2	ENSRNOG00000024523	-2.11397	0.041501
Kcne3	ENSRNOG00000017054	-1.56669	0.041586
Trim15	ENSRNOG00000031689	-1.19633	0.041993
Padi1	ENSRNOG00000007067	-6.43572	0.04203
Tmem252	ENSRNOG00000025476	-1.36181	0.042127
Esrrg	ENSRNOG00000002593	-2.41349	0.042554
Adcyap1r1	ENSRNOG00000012098	1.445273	0.043007
Ptchd1	ENSRNOG00000003675	-1.59409	0.043095
AABR07003250.2	ENSRNOG00000053819	-2.08925	0.043149
Esrp2	ENSRNOG00000023177	-1.5125	0.043327
Cpeb4	ENSRNOG00000033169	-1.41514	0.043619
Hsd17b14	ENSRNOG00000020949	-2.22592	0.043746
Myo5b	ENSRNOG00000014104	-1.58055	0.043817
Spata17	ENSRNOG00000002504	-1.1703	0.043979
Duox1	ENSRNOG00000033348	-2.63146	0.044047
Armc3	ENSRNOG00000016716	-1.87853	0.044095
Padi2	ENSRNOG00000007574	-1.83239	0.044117
Smim22	ENSRNOG00000042344	-1.3462	0.044193
Clec12b	ENSRNOG00000053638	-1.74776	0.044314
Isg20	ENSRNOG00000054561	-1.83614	0.044324
AC117905.2	ENSRNOG00000061352	2.571403	0.044585
Mir207	ENSRNOG00000035596	-2.74372	0.044697
LOC100911730	ENSRNOG00000019215	-3.15522	0.045167
Postn	ENSRNOG00000012660	-1.79995	0.045443
Ptgdrl	ENSRNOG00000031535	-6.36696	0.045725

Gm26247	ENSRNOG00000057316	-1.27329	0.045966
Sfn	ENSRNOG00000033153	-2.09161	0.046015
Fam81a	ENSRNOG00000057501	-2.1644	0.046035
Ppp1r15a	ENSRNOG00000020938	-1.06454	0.046244
Camk2b	ENSRNOG00000052080	1.21027	0.046283
Lrguk	ENSRNOG00000008775	-2.46214	0.046473
Kcnh3	ENSRNOG00000057315	-2.98093	0.046547
Ube2b	ENSRNOG00000059348	-1.68549	0.046637
Yif1b	ENSRNOG00000020616	1.855194	0.047062
7SK	ENSRNOG00000059596	-4.7686	0.047222
AABR07024907.1	ENSRNOG00000051631	-2.88327	0.047243
Swt1	ENSRNOG00000032258	-1.4412	0.047272
Psapl1	ENSRNOG00000006845	-5.3507	0.047521
Tacstd2	ENSRNOG00000007740	-2.57645	0.047965
Rbp2	ENSRNOG00000013932	-2.66374	0.048052
Esr1	ENSRNOG00000019358	-1.0264	0.048071
Sprn	ENSRNOG00000018927	-1.31315	0.048149
Gzmk	ENSRNOG00000010661	1.70136	0.048175
Fam161a	ENSRNOG00000009881	-1.6119	0.048191
Sfxn2	ENSRNOG00000059312	3.283071	0.048406
LOC499240	ENSRNOG00000013713	-5.47347	0.048509
Spp1	ENSRNOG00000043451	-1.42843	0.04851
Barx2	ENSRNOG00000008592	-3.73269	0.048787
LOC100911374	ENSRNOG00000001756	-2.35358	0.048817
Rfx2	ENSRNOG00000045846	-1.44444	0.048917
Tnfsf15	ENSRNOG00000008930	3.205743	0.04903
7SK	ENSRNOG00000058555	-3.06059	0.049106
Hbegf	ENSRNOG00000018646	-1.30224	0.049195
Cacybp	ENSRNOG00000002572	-1.41638	0.049454
Cdhr1	ENSRNOG00000013330	-1.8471	0.049633
Zfp707l1	ENSRNOG00000043439	1.644636	0.049666
Muc15	ENSRNOG00000004703	-3.56722	0.04968
Dydc2	ENSRNOG00000039591	-6.29477	0.049868
Ccdc146	ENSRNOG00000012932	-2.0915	0.049963

Table S2. Differentially expressed genes in ovaries from TBT + RSV-treated rats compared to the TBT rats. This table lists all genes detected on differential expression analysis from RNA-seq experiments at a cutoff of $\log_2$ (fold change) < - 1 or > 1 and $q \leq 0.05$.

Gene name	Gene ID	log2FoldChange	*P* value
Hnrnpab	ENSRNOG00000003645	-2.35136	4.58E-06
LOC103690005	ENSRNOG00000045655	-3.95421	7.89E-06
Ano4	ENSRNOG00000006841	2.013744	9.76E-06
RT1-DMb	ENSRNOG00000049491	5.723567	1.02E-05
LOC103689949	ENSRNOG00000021731	-3.03994	2.53E-05
Miox	ENSRNOG00000008694	-3.00011	5.31E-05
LOC100912538	ENSRNOG00000050877	3.056847	6.49E-05
LOC108348062	ENSRNOG00000040153	2.479533	7.97E-05
Hspb1	ENSRNOG00000023546	1.029936	8.13E-05
Hba-a1	ENSRNOG00000029886	1.406413	9.06E-05
Phf6	ENSRNOG00000048650	-5.05607	0.000103
Alas2	ENSRNOG00000000167	1.695616	0.00019
Epb41l1	ENSRNOG00000055809	3.107024	0.000249
Echs1	ENSRNOG00000047565	5.179755	0.000292
Nxpe5	ENSRNOG00000031343	3.821635	0.000321
Htr2a	ENSRNOG00000010063	2.965496	0.000506
Coa8	ENSRNOG00000011542	3.045231	0.000621
AC117330.1	ENSRNOG00000018925	-1.181	0.000721
Hbb	ENSRNOG00000058105	1.059845	0.001206
Impad1	ENSRNOG00000027079	-2.94177	0.001244
LOC108348106	ENSRNOG00000047943	3.938661	0.001257
LOC103693999	ENSRNOG00000019346	3.332146	0.001331
Kansl2	ENSRNOG00000053541	1.422231	0.001362
LOC103694855	ENSRNOG00000060629	3.277229	0.001414
LOC103694865	ENSRNOG00000054871	-1.40109	0.001593
Rbm39	ENSRNOG00000019848	1.617502	0.001636
NEWGENE_1308612	ENSRNOG00000059891	-2.36893	0.001655
Srm	ENSRNOG00000046379	-3.01328	0.001732
Abl1	ENSRNOG00000009371	2.18812	0.00284
Ccnb3	ENSRNOG00000002951	-1.20014	0.003206
Sftpc	ENSRNOG00000011177	-2.51761	0.003442
M6pr	ENSRNOG00000014992	1.508265	0.003699
Cenpa	ENSRNOG00000032178	-1.21113	0.003779
Pnliprp2	ENSRNOG00000017982	2.902986	0.004246
Coa8	ENSRNOG00000058920	-4.31945	0.004695
AABR07034586.1	ENSRNOG00000053105	4.56291	0.005033
Trpv2	ENSRNOG00000003104	1.548691	0.005464
Defa11	ENSRNOG00000038133	4.967935	0.005727
Clec12a	ENSRNOG00000054860	1.148279	0.006652
AC127784.5	ENSRNOG00000061943	-1.4051	0.006992
LOC100911440	ENSRNOG00000049944	-2.15042	0.007111
Baat	ENSRNOG00000007395	-3.56353	0.007518
Rec114	ENSRNOG00000009332	2.358158	0.007573

Atn1	ENSRNOG00000049575	-1.46921	0.008687
LOC103690317	ENSRNOG00000060185	-3.87657	0.008876
LOC100911672	ENSRNOG00000060594	-1.53703	0.009344
Polr2h	ENSRNOG00000001748	-3.46218	0.009781
Rergl	ENSRNOG00000008130	1.243645	0.01002
AABR07043200.1	ENSRNOG00000058276	-1.92085	0.010428
LOC100134871	ENSRNOG00000061299	1.832336	0.011731
AABR07026012.1	ENSRNOG00000022358	1.32575	0.012233
AABR07064000.1	ENSRNOG00000029594	-1.40729	0.012479
AC131537.2	ENSRNOG00000046410	-4.7521	0.012564
Fam50a	ENSRNOG00000045790	2.849997	0.013176
NEWGENE_1359268	ENSRNOG00000046920	2.766871	0.013453
AC141521.1	ENSRNOG00000053147	-2.22948	0.013729
Cldn2	ENSRNOG00000054495	1.404357	0.013832
LOC100910308	ENSRNOG00000046308	-1.77806	0.014133
Aspn	ENSRNOG00000050431	2.806795	0.015211
Sfxn2	ENSRNOG00000059312	-3.31748	0.015261
Fabp6	ENSRNOG00000003902	-1.03908	0.015654
Hs3st3a1	ENSRNOG00000024591	-2.35711	0.016032
Ncbp2	ENSRNOG00000001746	-2.14489	0.016039
LOC108348110	ENSRNOG00000060202	4.704119	0.016143
Htr1b	ENSRNOG00000013042	-1.84932	0.016167
LOC100909599	ENSRNOG00000059898	-2.17825	0.016543
AABR07027088.1	ENSRNOG00000046068	-2.70617	0.016594
Rtl9	ENSRNOG00000049853	4.579325	0.016718
AABR07066693.1	ENSRNOG00000047003	2.15985	0.017117
AABR07042611.1	ENSRNOG00000045837	-2.09495	0.017292
AABR07057233.2	ENSRNOG00000057241	-4.41728	0.017439
Ckm	ENSRNOG00000016837	-1.71095	0.017503
Nr1i2	ENSRNOG00000002906	3.314749	0.017851
Rmnd5b	ENSRNOG00000047396	-2.18485	0.017873
Faim	ENSRNOG00000030463	2.542631	0.018509
LOC108348065	ENSRNOG00000047281	1.173927	0.019198
Cdkn2a	ENSRNOG00000059837	1.40859	0.019843
Epha8	ENSRNOG00000013036	-4.79878	0.019977
LOC108348072	ENSRNOG00000047864	3.615451	0.020356
Ccdc142	ENSRNOG00000022326	-1.96464	0.020842
LOC100911881	ENSRNOG00000050181	-2.17845	0.020879
Myom3	ENSRNOG00000032994	-1.1042	0.020939
Fgf9	ENSRNOG00000011471	-1.02705	0.021142
Ndufa1	ENSRNOG00000040005	2.452556	0.021684
Zfp709l1	ENSRNOG00000048910	2.638614	0.022554
LOC500331	ENSRNOG00000030522	-4.783	0.022709
Aard	ENSRNOG00000004708	-1.0899	0.024069

LOC100912578	ENSRNOG00000059883	1.698422	0.024173
LOC688553	ENSRNOG00000037632	3.910385	0.025306
Usp18	ENSRNOG00000037198	1.018331	0.025587
Atf3	ENSRNOG00000003745	1.046146	0.025615
Marchf10	ENSRNOG00000007084	-2.24974	0.025647
Rln1	ENSRNOG00000060867	-2.4937	0.025921
Hbegf	ENSRNOG00000018646	-1.12754	0.025955
Ube2b	ENSRNOG00000059348	-1.57197	0.025999
Ms4a6e	ENSRNOG00000050395	-2.36738	0.026125
LOC103690026	ENSRNOG00000062153	-5.09159	0.026875
AABR07058658.1	ENSRNOG00000032311	1.011194	0.027312
Dntt	ENSRNOG00000013615	3.208157	0.027489
Foxq1	ENSRNOG00000021752	2.428267	0.02776
LOC108348114	ENSRNOG00000047573	-1.61603	0.027833
AABR07043523.1	ENSRNOG00000047845	-3.61155	0.027889
Chchd10	ENSRNOG00000028356	1.172863	0.027996
Ppef2	ENSRNOG00000052061	1.835565	0.028317
Adamts17	ENSRNOG00000037080	-1.01503	0.028343
Arntl2	ENSRNOG00000001830	2.698786	0.028346
Hmgb3	ENSRNOG00000011096	-1.79346	0.028415
Ctsw	ENSRNOG00000027096	-1.30532	0.028531
Tlr11	ENSRNOG00000032368	1.150307	0.028644
Atoh7	ENSRNOG00000000384	-2.41153	0.028869
Car7	ENSRNOG00000012371	2.866834	0.029136
LOC100911548	ENSRNOG00000015125	2.942157	0.029564
Chgb	ENSRNOG00000021269	-1.73222	0.030393
AABR07044415.1	ENSRNOG00000055766	-3.79923	0.030865
LOC100911625	ENSRNOG00000048365	1.861031	0.031585
AABR07013464.1	ENSRNOG00000023965	3.103134	0.031718
Fam111a	ENSRNOG00000012067	1.894154	0.032613
Abhd10	ENSRNOG00000054334	1.909422	0.033858
AABR07029452.1	ENSRNOG00000058208	-1.60894	0.034162
Batf3	ENSRNOG00000003716	1.334021	0.034574
AABR07003049.1	ENSRNOG00000037364	-3.8604	0.034743
Klk14	ENSRNOG00000033706	2.217266	0.034858
Oas2	ENSRNOG00000049282	1.020798	0.035066
Rufy4	ENSRNOG00000032991	1.717218	0.035209
Col10a1	ENSRNOG00000051399	-1.73694	0.035929
Fam205a	ENSRNOG00000042596	1.162478	0.036271
Prmt8	ENSRNOG00000053804	-3.01474	0.036358
Rnase11	ENSRNOG00000039543	-3.91247	0.037239
Rnase12	ENSRNOG00000042612	4.233041	0.037298
B3gnt3	ENSRNOG00000018764	-1.94746	0.037966
Gpat2	ENSRNOG00000013906	-1.61754	0.039419

AABR07000738.1	ENSRNOG00000052999	-1.33638	0.040045
Ca8	ENSRNOG00000005669	1.525418	0.040093
Csmd1	ENSRNOG00000030719	1.084213	0.040718
Clec4m	ENSRNOG00000029881	-4.37026	0.041522
Tmco3	ENSRNOG00000046973	2.388428	0.04168
LOC100911374	ENSRNOG00000001756	2.664253	0.041917
Fras1	ENSRNOG00000002053	-1.14879	0.041934
Orm1	ENSRNOG00000007886	-1.47266	0.04227
AABR07017208.2	ENSRNOG00000061006	4.655646	0.042295
Tmem196	ENSRNOG00000037435	1.882982	0.042322
Lrfn5	ENSRNOG00000005550	2.531082	0.042333
Col26a1	ENSRNOG00000001422	-2.76534	0.042539
LOC103689945	ENSRNOG00000009596	1.048767	0.044627
Fcer2	ENSRNOG00000001005	-1.81642	0.04469
Sytl2	ENSRNOG00000030776	2.453091	0.045421
Mcart1	ENSRNOG00000039278	-1.23895	0.045698
Coch	ENSRNOG00000005286	-1.49831	0.046317
Clec4n	ENSRNOG00000010057	-3.59239	0.047194
Aqp3	ENSRNOG00000009797	-1.33164	0.047913
Cpa1	ENSRNOG00000010725	-2.37574	0.048084
C1qtnf4	ENSRNOG00000009140	-1.91394	0.048508
Plch1	ENSRNOG00000009955	1.354005	0.048547
Tdrd9	ENSRNOG00000053631	-2.06777	0.048979
AABR07045071.1	ENSRNOG00000061091	1.824412	0.049035
Btnl7	ENSRNOG00000048841	-2.85058	0.049254
C1qtnf3	ENSRNOG00000018570	-2.73196	0.049539

Table S3. Primers for the genes analyzed in rat ovaries.

Gene Number	Gene symbol	Sequence (5'--3')	Product length (bp)
NM_017254.1	*Htr2a*	F: GTTGATGACTGCTCCATGGTT	85
		R: CCTTTTCATTCACGGTTTCAA	
NM_001191707.1	*Plch1*	F: ACACCTGAAGCAGAACCTGG	75
		R: CCAAAGTTGGCCATCAGGGA	
XM_008771394.2	*Tmco3*	F: AGCTGCTGGACGTCTCAATG	240
		R: GCACTGCCAACACAAACTTC	
NM_001106165.1	*Car7*	F: CGAGCGGACAGAACGGAG	105
		R: CAATTTGAAGGGCCGTCGT	
NM_001042354.1	*Camk2b*	F: TGCAAGGAGGAAGCTCAAGG	117
		R: CTGTTTGTCTGGGGCTTGAC	
NM_012912.2	*Atf3*	F: GACAGACAGCCCGCCTCTA	317
		R: CTTCAGGGGCCACCTCAGAC	
NM_001037769.2	*Coa8*	F: CGAACCGAGTCAGGCCAAAGA	255

		R: CTCGTAAATGGACCTGGTGCT	
XM_039088894.1	*Cfap44*	F: ATGGGGAGGTCTTAGCCACT	146
		R: AGTGCTTTCAGGGTGACTGT	
NM_057191.2	*Klhl41*	F: GGGCGGAAAGACAGATGACA	225
		R: TCCGTCATCACTTCCCACTTAT	
NM_001012116.1	*Spag1*	F: GTTCGCACGGGAGTGGAAA	289
		R: GCCTTCTTCACCAGACCTTAG	

Table S4. Clinical characteristics in women with or without PCOS.

PCOS: polycystic ovary syndrome; BMI: body mass index; FSH: follicle stimulating hormone; AMH: anti-Müllerian hormone; AFC: antral follicle counting.

	Cycle ID	Age (years)	BMI (kg/m^2)	FSH (IU/L)	AMH (pg/L)	AFC
Control	211446	28	20.96	5.53	5.87	19
Control	211447	30	21.03	5.45	7.2	22
Control	211486	27	23.71	4.61	7.88	24
PCOS	211453	27	21.34	3.97	3.43	24
PCOS	211456	37	24.3	6.38	4.94	24
PCOS	211465	34	25.39	8.2	11.8	20
PCOS	211472	27	21.72	4.44	10.9	24
PCOS	211481	27	21.31	7.34	13.6	21
PCOS	211542	27	23.83	6.89	5.37	24
PCOS	211545	30	22	2.38	4.99	18
PCOS	211572	30	20.83	7.47	15.63	21
PCOS	211645	24	24.74	6.01	7.16	24
PCOS	211685	32	23.69	7.12	13.9	24
PCOS	211694	29	21.46	6.71	23	24
PCOS	211743	35	23.73	9.22	10.08	24
PCOS	211752	27	19.56	2.65	6.34	24
PCOS	211754	31	19.38	5.21	8.25	18
